# A Magnetic Force Microscopy Study of Patterned T-Shaped Structures

**DOI:** 10.3390/ma14061567

**Published:** 2021-03-23

**Authors:** Elis Helena de Campos Pinto Sinnecker, José Miguel García-Martín, Dora Altbir, José D’Albuquerque e Castro, João Paulo Sinnecker

**Affiliations:** 1 Instituto de Física, Universidade Federal do Rio de Janeiro, Caixa Postal 68528, Rio de Janeiro 21941-972, Brazil; elis@if.ufrj.br (E.H.d.C.P.S.); jcastro@if.ufrj.br (J.D.eC.); 2 Instituto de Micro y Nanotecnología, IMN-CNM, CSIC (CEI UAM+CSIC), Isaac Newton 8, 28760 Tres Cantos, Spain; 3 Departamento de Física, CEDENNA, Universidad de Santiago de Chile (USACH), Avda. Ecuador 3493, Santiago, Chile; dora.altbir@usach.cl; 4 Centro Brasileiro de Pesquisas Físicas—CBPF/MCTIC, Rua Dr. Xavier Sigaud 150, Urca, Rio de Janeiro 22290-180, Brazil; sinnecker@cbpf.br

**Keywords:** Magnetic Force Microscopy, Micromagnetic Simulations, magnetic materials, data storage and retrieval, electron beam lithography

## Abstract

The study of patterned magnetic elements that can sustain more than one bit of the information is an important research line for developing new routes in magnetic storage and magnetic logic devices. Previous Monte Carlo studies of T-shaped magnetic nanostructures revealed the equilibrium and evolution of magnetic states that could be found as a result of the strong configurational anisotropy of these systems. In this work, for the first time, such behavior of T-shaped magnetic nanostructures is experimentally studied. In particular, T-shaped Co nanostructures have been produced by electron beam lithography using a single step lift-off process over Si substrates. The existence of four magnetic stable states has been proven by Magnetic Force Microscopy (MFM) and the analysis was complemented by Micromagnetic Simulations. The results confirmed that even for what can be considered large structures, with μm sizes, such four stable magnetic states can be achieved, and therefore two magnetic bits of information can be stored. We also addressed how to write and read those bits.

## 1. Introduction

The study of patterned magnetic elements that can sustain more than one bit of the information is an important research line for developing new routes in magnetic storage and magnetic logic devices [1,2]. The most popular systems in the literature in this line are those with flat shape that are able to exhibit flux-closure states, such as square and diamond elements in the Landau configuration with four equivalent domains [3,4,5] and circular elements exhibiting a vortex state [6,7]: one bit is associated with the sense of rotation of the magnetization in the plane of the element, clock wise or counter-clock wise, whereas the other is associated with the magnetic moments in the core (center) that are vertically aligned pointing up or down. However, there are several issues to deal with these systems in a real application: changing the magnetic state at the core without altering the in-plane magnetization, i.e., by applying a pure vertical magnetic field, requires high field values [8,9], while changing the in-plane magnetization rotation at will demands introducing a morphological asymmetry [10,11,12]. Another approach consists of fabricating magnetic elements with tailored shapes that induce configurational anisotropy. This is the case of triangles and pentagons [13,14], but replicating such shapes in large arrays for a real application would make those systems extremely challenging. A few years ago, Escobar and co-workers published a couple of theoretical works proposing T-shaped elements in the x-y plane to store two bits [15,16], with each branch contributing with one bit: the magnetization in each rectangle would be aligned parallel to the long side, in the positive direction or in the negative one. As a result, if the reduced magnetization of the rectangle aligned with the *i* axis is given by *m*_i_ = *M*_i_/*M*_s_, where *M*_i_ is the magnetization along the *i* axis and *M*_s_ is the saturation magnetization of the rectangle, there are four stable configurations for T-shaped elements, which labelled as [*m*_x_, *m*_y_] are: [1, 1], [−1, 1], [1, −1] and [−1, −1]. In this work, those T-shaped elements have been fabricated with e-beam lithography and the existence of these four magnetic states has been studied with Magnetic Force Microscopy (MFM) and by Micromagnetic Simulations.

## 2. Materials and Methods

**Figure 1:**
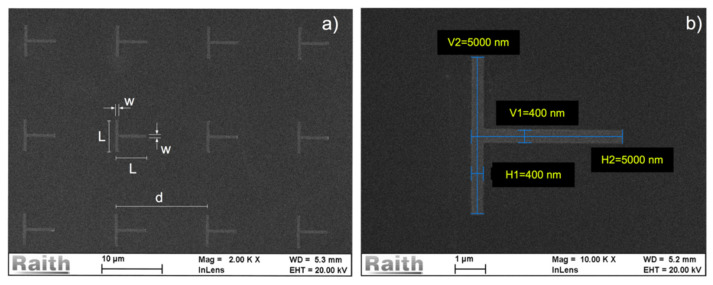
(**a**) SEM image of the array of T-shaped structures after lift-off showing the arm length L = 5 μm, arm width w = 400 nm and inter structure distance d = 15 μm. (**b**) Dimensions H1 = 400 nm, V1 = 400.0 nm, H2 = 5000 nm, V2 = 5000 nm of one individual T-shaped structure after lift-off measured with SEM.

Regular arrays of T-shaped Co nanostructures have been fabricated by electron beam lithography using a single step lift-off process over Si substrates with 3000 nm SiO_2_ layer. The virgin Si/SiO_2_ substrates were previously cut to 2 × 2 cm^2^ square pieces and cleaned with acetone, isopropanol, and DI Water in ultrasound bath. A single layer of positive commercial AllResist ARP-672.045 PMMA/950K was deposited at 4000 RPM using a WS-650-23 Laurell spinner (Laurell Technologies Corporation, North Wales, PA, USA), resulting in a 230 nm thick PMMA layer. The substrate with the PPMMA has been prebaked at 150 °C in a hot plate previous to the e-beam exposure. The array nanostructures were designed in a GDSII file and exposed after careful dose test in order to tune the appropriate dose for each aspect ratio structure. The initial dose has been chosen according to the ARP-672.045 data sheet (95 μC/cm^2^) and fine-tuned to deliver the best lithographed structures. The final doses were in the 90 μC/cm^2^–110 μC/cm^2^ range. The exposures were performed in a Raith e-Line nanolithography system at the LABNANO/CBPF. After the exposure, the structures were developed in a 7:3 Isopropanol/ Deionized water solution [17,18] and carefully dried with Nitrogen flux. Metallic films were subsequently deposited using DC magnetron sputtering in an AJA International sputtering system (AJA International Inc., North Scituate, MA, USA), equipped with A300-XP magnetrons, at a base pressure of the order of E-08 Torr. The T-shaped structures studied in this work are metallic films stacks of Ta (1 nm)/Co (33 nm)/Ta (1 nm) with arm length L = 5 μm, arm width w = 400 nm, film thickness t = 35 nm, and structure separation d = 15 μm. The T-shape structure dimensions were chosen in order to guarantee an in-plane monodomain magnetization along each of the T-shape arms [19,20]. The 1 nm Ta buffer over the Si improves the Co layer adhesion and the Ta 1nm cap layer over the Co avoids oxidation. The deposition conditions were 5 mTorr pressure, 50 sccm Ar gas flow, and 150 mA on the DC magnetron current. The lift-off has been performed under acetone and ultrasound bath. All film thicknesses were calibrated using X-Ray reflectivity [21] in small angle incidence in using a PANalytical X’Pert PRO diffractometer (Malvern Panalytical Ltd, Malvern, UK) with Cu Kα1 X-rays with 1.54056 Angstrom wavelength. Figure 1 shows the scanning electron microscopy (SEM) images of the Co T-shaped structures after the lift-off process.

Magnetic Force Microscopy (MFM) experiments were performed using a Dimension Icon microscope (Bruker Corporation, Billerica, MA, USA) operating at ambient conditions. The magnetic probes were MESP model from Bruker (main characteristics: CoCr coating, force constant about 3 N/m, resonant frequency about 75 kHz and tip radius about 35 nm). According to Jaafar et al. [22], the coercivity of those probes is 65 mT and they were successfully used to image commercial hard disks (i.e., samples with high stray field). The double-pass scanning method in tapping mode was used: the topography of the surface along one line of the image was acquired during the first pass, then the tip was lifted 60 nm above the sample and the scan line was repeated during the second pass (thus ensuring that the tip-to-sample distance was always 60 nm) and the phase shift induced by the magnetic interaction between tip and sample was recorded. This phase shift is proportional to the force gradient, and as the tip was magnetized prior to the experiments in the vertical direction, i.e., perpendicular to the sample surface, the obtained magnetic contrast can be ascribed to the magnetic poles at the sample surface. It is worth noticing that electrostatic interaction between the tip and sample can be neglected when compared to the magnetic interaction between them, since the structures to be analyzed are ferromagnetic and exhibit high magnetic moment. This is in clear contrast to what happens in samples with low magnetic moment, such as superparamagnetic nanoparticles, magnetic oxide nanowires and carbon-based materials, which require strategies to separate the interactions and compensate the electrostatic one [23,24,25].

We performed micromagnetic simulations using the Mumax3 [26] open software that calculates the space- and time-dependent magnetization in nano- to micro-sized ferromagnets using a finite-difference discretization. We performed only static simulations that are obtained by an energy minimization method. The simulations were performed on individual T-shaped structures with L = 5 μm. As in the lithographed samples with L = 5 μm the individual structures are separated by d = 15 μm, the magnetic dipolar interactions among T-shaped structures can be neglected and we can simulate only one individual structure. The calculations were carried out with the following co-parameters: saturation magnetization *M*_s_ = 1700 × 10^3^ A/m and exchange stiffness *A* = 30 × 10^−12^ J/m [12,27,28]. According to these parameters, the exchange length of the Co film is of the order of 5 nm, and therefore we used a 5 nm cell size in all directions (x, y and z). As we were interested only in stable states, and not in the dynamics, the Gilbert damping was set to α = 0.1.

## 3. Results

**Figure 2:**
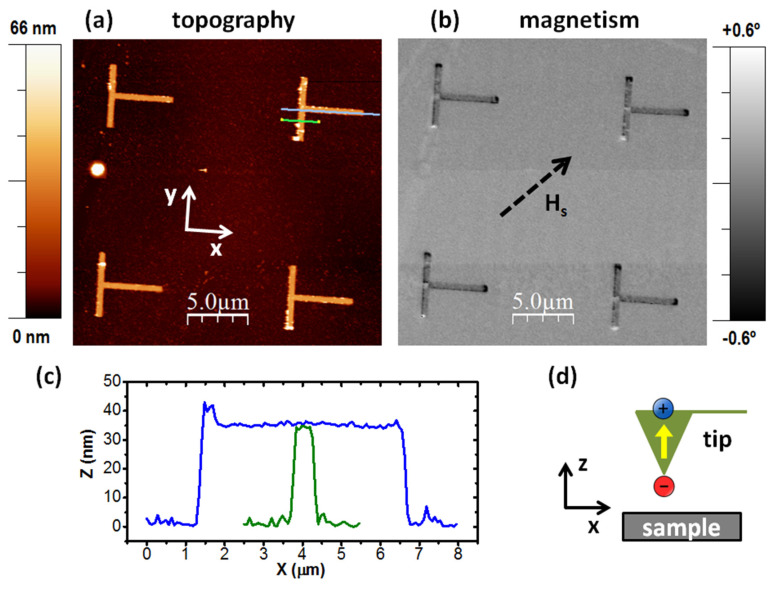
(**a**) Topographic and (**b**) magnetic images, respectively, of four T-shaped structures. (**c**) Topographic profiles along the blue and green lines painted in (**a**). (**d**) Scheme of the magnetic state of the tip.

The MFM experiments were performed with the L = 5 μm T-shaped structures in the remanent state obtained after the application of a saturating magnetic field in a given in-plane direction. Such field was applied ex situ with an electromagnet. Figure 2a,b show the topographic and magnetic images, respectively, of four T-shaped structures, with the previously applied magnetic field direction indicated by the black dashed arrow in Figure 2b. In the case of the topographic image, we subtracted a constant to each scan line of the unprocessed image to remove the low frequency noise that appears as an irregular distribution of the scan height; the areas with inherent different height, such as the T-shaped structures and the leftovers of the resist on the substrate, were removed from the calculation of the constant. However, for the magnetic image, the unprocessed image was not altered but for the proper adjustment of the gray scale, in this case covering a phase range of 1.2°. Figure 2c shows the topographic profiles obtained along the green and blue lines painted in Figure 2a: the expected 35 nm thickness, 400 nm arm width, and 5 μm arm length is experimentally verified. The magnetic image in Figure 2b was obtained with the tip of the microscope prepared in the magnetic state depicted in the scheme in Figure 2d, i.e., with magnetization pointing along the normal to the sample surface and with the south pole (negative magnetic charge) at the tip apex. This preparation helps identify the magnetic poles at the structures: magnetic attraction will be due to north poles (positive magnetic charges) at the sample, whilst repulsion will be due to south poles (negative magnetic charges). These positive and negative magnetic charges appear with dark and bright contrast, respectively, with the gray scale used in our case, which is also shown in Figure 2b. It is important to notice in Figure 2b that all the T-shaped structures exhibit the same magnetic state, which confirms the homogeneity of the fabricated sample. This state is one of the four magnetic states analyzed in detail in Figure 2.

**Figure 3:**
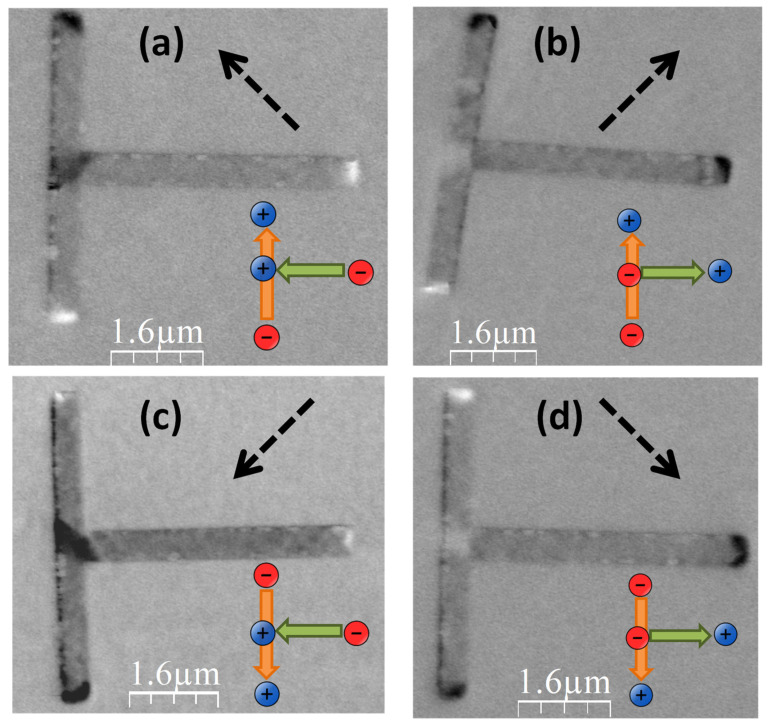
Magnetic Force Microscopy (MFM) images of the four different remanent states obtained in T-shaped structures after saturating them with fields applied along the four directions depicted with dashed black arrows. The arrow insets show the orientation of the magnetization in the two branches of the T in each case. Labelled as [*m*_x_, *m*_y_]: (**a**) [−1, 1] (**b**) [1, 1] (**c**) [−1, −1] (**d**) [1, −1].

Figure 3 shows the MFM images obtained after the saturation (ex situ, with an electromagnet) of the T-shaped structures with fields applied along the four directions depicted with dashed black arrows. Again, the only alteration of the acquired images is the proper adjustment of the gray scale to highlight the magnetic features, covering a phase range between 1.3° and 1.6°. Depending on the direction of the applied field, one can put each structure in four different remanent states. Taking into account the bright and dark contrast obtained in each case, as well as the discussion above about its meaning, the arrow insets in Figure 3 indicate the magnetization direction in the two branches of the T-shaped structures. Labelled as [*m*_x_, *m*_y_], those four magnetic states are: [−1, 1] in Figure 3a, [1, 1] in Figure 3b, [−1, −1] in Figure 3c, and [1, −1] in Figure 3d. Two subtle details in all these images can be discussed:

(i) There is a global attractive force between the tip of the microscope and the T-shape structures in the experiments. Excluding the four regions that have a large accumulation of magnetic poles (i.e., the three extreme points of the T-shape and the point of intersection between the two branches), the structure exhibits a slightly darker contrast than the substrate (which is obviously a zone without magnetic interaction with the tip).
(ii) In the four regions with large accumulation of magnetic poles, the apparent size of the dark regions is larger than that of the bright ones.

Both effects are reversible phenomena induced by the tip of the MFM equipment [4,29]. What happens in (i) is due to the small distortion of the magnetic moments of the sample produced by the tip stray-field, which tends to align slightly these moments along its direction. Regarding (ii), in those four regions the magnetic pole at the tip apex is attracting or repelling the opposite or similar poles of the sample, respectively, and thus enlarging or shrinking those regions during the experiment.

**Figure 4:**
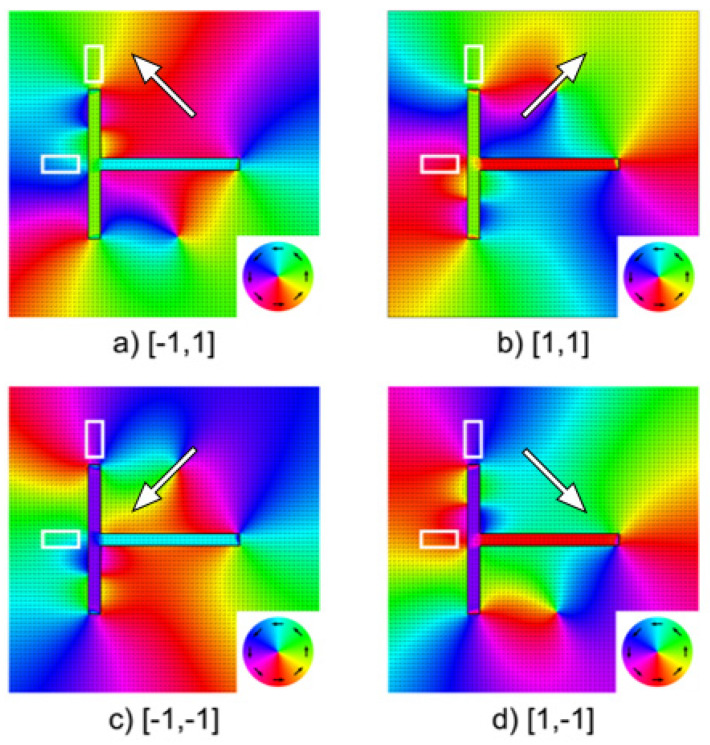
Micromagnetic simulations of the four relaxed stable magnetic states [*m*_x_, *m*_y_] at H = 0 (remanent state) for T-Shapes with L = 5 μm and the stray-field generated by them: (**a**) [−1, 1], (**b**) [1, 1], (**c**) [−1, −1] and (**d**) [1, −1]. The white arrows indicate the saturating H field direction applied before the structures are relaxed. The circular insets represent the colormap proportional to magnetic flux induction direction on the xy plane, also indicated by the black arrows. The hollow rectangles indicate areas that can be inspected by tunnel magnetoresistance (TMR) heads in order to read the stray fields generated by the recorded magnetic states and recover the information.

**Figure 5:**
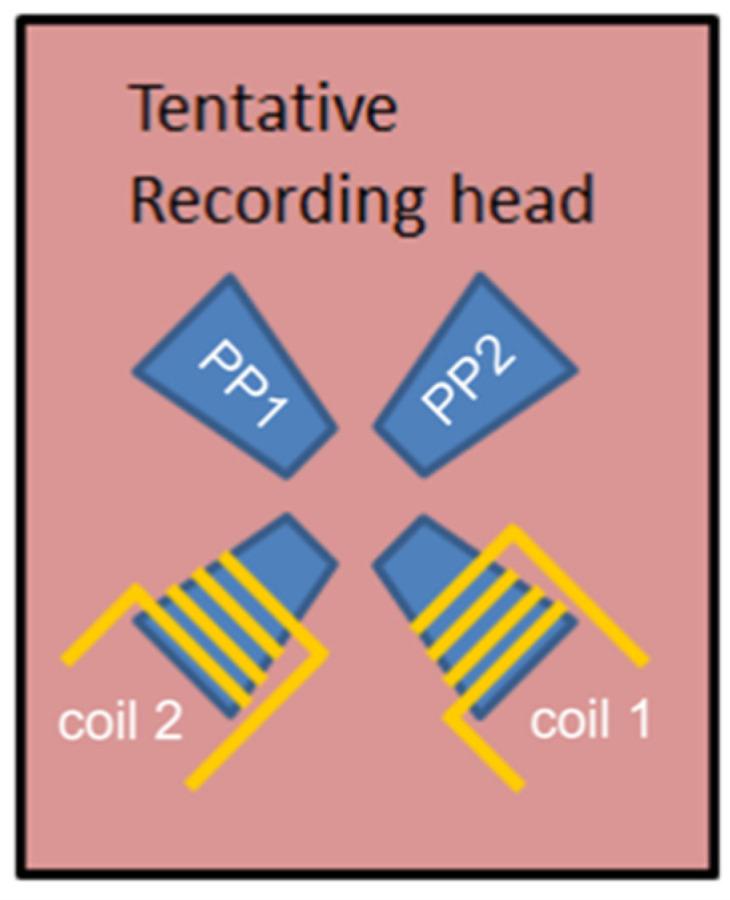
Possible device for recording the magnetic states of an individual T-shaped structure. It consists of two magnetic pole pieces, PP1 and PP2 assembled perpendicular to each other, and energized by individual coils, coil 1 and coil 2. The head is assembled as to produce the magnetic field in the [1, 1]/[−1, −1] or in the [−1, 1]/[1, −1] directions.

Micromagnetic simulations were performed considering a T-shaped structure with the same geometrical dimensions than that experimentally analyzed by MFM. Figure 4 shows the final magnetic configurations of such L = 5 μm, w = 400 nm, t = 35 nm T-shaped simulated structure. The structure was first magnetized with a saturating field applied in the direction indicated by a big white arrow. Afterwards, the field was removed, and the magnetization of the structure was let to relax. The final state in each case corresponds to the same four states experimentally found, pinpointing that those four states are minima in the energy landscape. Each of these four states could be recorded using a magnetic recording head as shown schematically in Figure 5, which would allow for switching individual structures. The head consists of two magnetic pole pieces, PP1 and PP2 assembled perpendicular to each other, and energized by individual coils, coil 1 and coil 2. The head is assembled as to produce the magnetic field in the [−1, 1]/[1, −1] direction with coil1 or in the [1, 1]/[−1, −1] direction with coil2. Both coils would never be simultaneously energized.

**Figure 6:**
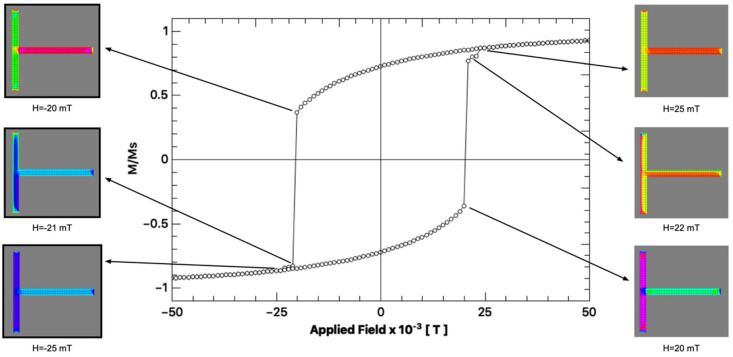
Simulated hysteresis loops a single T-shaped structure with L = 5 μm. The external field is applied along the [1, 1] direction and the magnetization is the total reduced magnetization along that direction. A sudden jump of the magnetization is observed both around H = −20 mT and H = 20 mT, the field at which both arms of the structure exhibit magnetization reversal.

From the micromagnetic simulation it is also possible to obtain the hysteresis loops of an individual structure and correlate its features to the stable states of the magnetization. Figure 6 shows the hysteresis loop calculated for one individual T-shaped structure. The external field is applied along the [1, 1] direction and the magnetization is the total reduced magnetization along that direction. When the field is being swept from positive to negative values, i.e., from the [1, 1] direction to the [−1, −1] direction, the structure changes from the [1, 1] stable state to the [−1, −1] state, producing a sudden jump in the hysteresis loop. The micromagnetic simulation shows a symmetric magnetic hysteresis loop with coercivity of around 20 mT, i.e., the change from the [1, 1] state to the [−1, −1] state occurs at 20 mT. The dynamics of the reversal process was also simulated and a video of it can be found in the Supplementary Material session.

## 4. Conclusions

We successfully fabricated T-shaped cobalt structures and studied their magnetic structure with MFM. Micromagnetic simulations were used to analyze the experimental results. It has been shown that even for what can be considered a large structure, with μm sizes, four stable magnetic states can be achieved, and therefore two magnetic bits of information can be stored. We also addressed how to write and read those bits.

## Data Availability

The data presented in this study are available upon reasonable request to the corresponding author.

## Supplementary Materials

The following material is available online at https://www.mdpi.com/1996-1944/14/6/1567/s1, Video S1: Reversal process of the T-Shaped structure when the field is being swept from the [1, 1] direction to the [−1, −1] direction, as a function of time. The initial state is the remanent state at H = 0. The field is then applied, at t = 1.000 ns, in the [−1, −1] direction with an intensity of H = −0.021 mT. The complete reversal process takes approximately 3.000 ns.
